# A Case of Appendiceal Pinworms in an Adolescent Patient

**DOI:** 10.7759/cureus.82714

**Published:** 2025-04-21

**Authors:** Zachary S Kauffman, David L Stuart

**Affiliations:** 1 Research, Lincoln Memorial University - DeBusk College of Osteopathic Medicine, Beckley, USA; 2 General Surgery, Beckley Appalachian Regional Hospital, Beckley, USA

**Keywords:** appendicitis, diagnostic laparoscopy, enterobiasis, enterobius vermicularis, pinworms

## Abstract

While pinworm infections are common among children and adolescents, pinworms mimicking appendicitis are relatively rare. Notably, such manifestations may present in the absence of typical signs and symptoms of appendicitis. The appendix may even appear normal on a computed tomography (CT) scan and upon laparoscopic examination. The current case report aims to expand the literature on appendiceal pinworms while also showcasing the diagnostic challenge that such cases may represent. A 15-year-old female presented to the emergency department with acute onset of right lower quadrant abdominal pain and vomiting. Initial imaging studies, including CT of the abdomen and pelvis, revealed a normal-appearing appendix and gallbladder. Initial laboratory examinations revealed anemia; leukocytosis was notably absent. Despite the unremarkable findings, the severity of her pain continued to increase, leading to the decision to perform a diagnostic laparoscopy with appendectomy. The procedure was uncomplicated, and the removed appendix appeared grossly normal. However, histological examination of the appendix revealed intraluminal *Enterobius vermicularis *without transmural inflammation. Medical management of pinworm infestations is relatively simple. However, appendiceal pinworm infestations may not be diagnosed until surgical intervention has been employed. Case reports such as these offer insight into the diagnostic challenge that may be posed by appendiceal pinworms, emphasizing that high clinical suspicion may be needed to avoid unnecessary surgery.

## Introduction

Helminthic infections, notably pinworm infections, are common among children and adolescents [1]. However, pinworm infestations mimicking appendicitis are rare. The incidence of acute appendicitis is about 100 cases per 100,000 people per year [2,3], making it a common cause of acute abdomen. With a mean age at presentation of 28 years [4], acute appendicitis is most common before or during early adulthood. Acute appendicitis is typically caused by obstruction of the appendiceal lumen. The etiology of this obstruction tends to differ between children and adults, with the most common etiology of acute appendicitis being lymphoid hyperplasia in children, whereas the most common etiologies of acute appendicitis in adults include infection, fecaliths, and tumors [4].

While acute appendicitis is typically a clinical diagnosis, the use of various laboratory examinations and imaging can aid in diagnosis, especially when the clinical picture is not clear. White blood cell (WBC) count and C-reactive protein (CRP) are the two most commonly utilized labs in the diagnosis of acute appendicitis. Specifically, both WBC count and CRP within the normal range have a high negative predictive value for acute appendicitis. On the other hand, most patients with acute appendicitis have a WBC count >10,000 cells/mm^3^, and a significantly increased WBC count >17,000 cells/mm^3^, in addition to elevated CRP, may indicate complicated appendicitis [5]. In terms of the role of imaging in acute appendicitis, a CT scan demonstrates >95% accuracy in the diagnosis of acute appendicitis in adults, and it is thus the preferred imaging technique. In populations in which ionizing radiation risk precludes the use of CT, such as in pregnant women and children, ultrasound may be efficacious. Finally, MRI may also be used to aid in the diagnosis of acute appendicitis, especially in pregnant women with equivocal ultrasound findings [4].

The most common complication of acute appendicitis is perforation, which can lead to abscess formation and peritonitis [4]. Complicated appendicitis is best evaluated with CT [6]. Signs of complicated appendicitis include abscess, wall enhancement defects, extraluminal air, and extraluminal appendicolith. These findings generally have a high specificity but low sensitivity [6]. Treatment options for appendicitis complicated by abscess or mass formation may include conservative medical management, percutaneous drainage with or without interval appendectomy, or emergency laparoscopic or open appendectomy. The best approach is somewhat controversial, though there is evidence that outcomes may be more similar than not across treatment modalities [7].

*Enterobius vermicularis*, or pinworms, is an uncommon cause of appendicitis. An estimated 2.7% of removed appendices include parasitic infestations, with over half of such cases having *Enterobius vermicularis* as the parasitic etiology [8]. *Enterobius vermicularis* typically affects those under age 18, and it is most common in males, with a male-to-female ratio of two-to-one. However, a female predominance is seen in children and adolescents, particularly those 14 years old and younger. Transmission is fecal-oral [9]. The pooled global prevalence of enterobiasis among children is estimated at 12.9%, while the weighted prevalence of enterobiasis among children in North America is about 1.9% [10].

About one-third of patients with *Enterobius vermicularis* are asymptomatic. The most common symptom is pruritus ani, or itching around the anus, which tends to be most common at night. Other common symptoms include perianal erythema, female genitourinary symptoms, and watery diarrhea. Less commonly, abdominal pain and appendicitis may result from worms blocking the appendiceal lumen [9]. In cases of *Enterobius vermicularis*, eosinophil count may be elevated [10]. In patients with *Enterobius vermicularis*-associated acute appendicitis, elevated CRP, leukocytosis, and neutrophilia are common [11].

Diagnosis of pinworms is established with the cellophane tape test [12], in which tape is placed on the anus to collect eggs in the morning. The cellophane tape test has a sensitivity of about 90% if samples are collected on three separate mornings [12]. Pinworms are treated medically with a single dose of either 100 mg of mebendazole, 11 mg/kg of pyrantel pamoate, or 400 mg of albendazole [9,12].

The current case report is unique in that it details a patient who presented clinically with symptoms of appendicitis despite an appendix that appeared normal both on CT scan and laparoscopy. Intraluminal pinworms were later discovered upon histological examination. The current case report serves to outline the case and to explore the diagnostic challenge associated with the diagnosis and management of appendiceal pinworms mimicking appendicitis.

## Case presentation

A 15-year-old female presented to the emergency department complaining of the acute onset of right lower quadrant abdominal pain, nausea, vomiting, and anorexia. She denied fever, chills, diarrhea, dysuria, oliguria, cough, or shortness of breath. Her past medical history and family history were noncontributory; she had no allergies, and her immunizations were up to date.

On physical examination, she was afebrile and hemodynamically stable with a temperature of 36.8°C, a pulse rate of 80/minute, and a respiratory rate of 18/minute. Cardiopulmonary auscultation was significant only for a chronic murmur. An abdominal exam revealed a soft and nondistended abdomen with normoactive bowel sounds and no masses. Tenderness to palpation was noted in the right lower quadrant.

Initial pelvic ultrasound revealed a thickened endometrium without pelvic free fluid. Two-view abdominal X-rays showed constipation without bowel obstruction, and computed tomography (CT) of the abdomen and pelvis with contrast revealed mild hepatomegaly, mild fecal retention, moderate hydrometra, and a 3.0 cm left ovarian cyst. Notably, the gallbladder and appendix were unremarkable. The next day, a kidney, ureter, and bladder (KUB) X-ray again showed fecal retention in the distal colon without obstruction and a distended stomach.

Laboratory examination revealed anemia and no leukocytosis. Laboratory investigations are included in Table 1.

**Table 1:** Laboratory evaluations of a 15-year-old female with abdominal pain revealing anemia but a lack of leukocytosis % - the relative percentage of total WBCs for that particular WBC; # - the absolute count of that WBC

Hematology	Patient Values	Reference Range	Units
WBC	4.4	3.9-11.6	×10^3^/uL
RBC	4.1	3.64-5.36	×10^6^/uL
Hgb	9.7	11.2-15.5	g/dL
Hct	31.2	32.9-46.2	%
Neut (%)	43.0	33.0-89.0	%
Lymph (%)	37.7	4.0-51.0	%
Mono (%)	16.8	3.0-15.0	%
Baso (%)	0.5	0.0-2.0	%
Eosin (%)	1.8	0.0-6.0	%
Neut (#)	1.9	1.5-7.1	×10^3^/uL
Lymph (#)	1.7	0.7-4.3	×10^3^/uL
Mono (#)	0.7	0.2-1.2	×10^3^/uL
Baso (#)	0.0	0.0-0.1	×10^3^/uL
Eosin (#)	0.9	0.0-0.8	×10^3^/uL
Chemistry			
Sodium	139.0	133-144	mEq/L
Potassium	4.0	3.5-5.0	mEq/L
Chloride	105.0	99-108	mEq/L
CO2	24.0	21-29	mEq/L
Anion Gap	11.0	5-15	mEq/L
BUN	11.0	5-18	mg/dL
Creatinine	0.6	0.3-0.9	mg/dL
AST	13.0	7-35	U/L
ALT	15.0	6-24	U/L
Alkaline Phosphatase	76.0	24-368	U/L

Differential diagnoses included acute appendicitis, constipation, mesenteric adenitis, or tubo-ovarian pathology.

**Figure 1:**
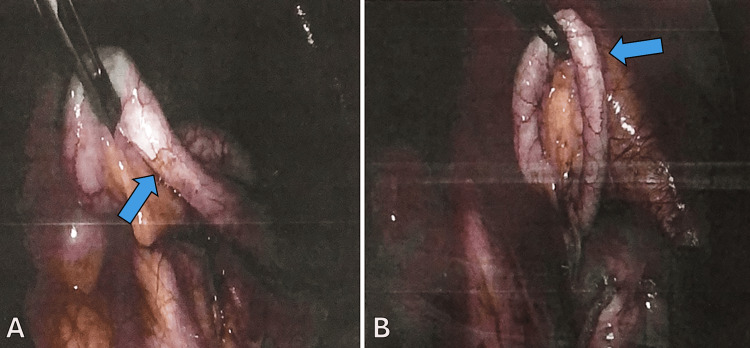
Laparoscopic images showing a normal-appearing appendix in a 15-year-old female with abdominal pain. The appendix is highlighted by a blue arrow.

The patient’s pain continued to worsen over the next two days, leading to the decision to proceed with diagnostic laparoscopy and appendectomy. The operative course was uncomplicated, and laparoscopy revealed a normal-appearing appendix (Figures 1A-1B), a large left-sided ovarian cyst, and a possible rupture of a right-sided ovarian cyst. The appendix was collected and sent to pathology for histological evaluation.

**Figure 2:**
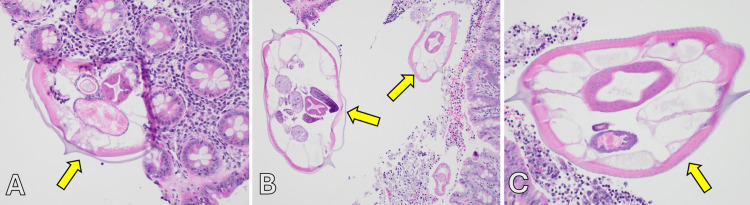
Histopathological examination from the appendix of a 15-year-old female showing Enterobius vermicularis in the lumen of the appendix. Enterobius vermicularis is highlighted by the yellow arrows.

Pathology revealed intraluminal adult *Enterobius vermicularis* (Figures 2A-2C) without transmural inflammation.

The patient recovered after surgery, and she was discharged home with MiraLAX (polyethylene glycol 3350), 17 g daily as needed, and albendazole, 400 mg once, with instructions to repeat in one week. However, she returned the same day complaining of 10 out of 10 abdominal pain localized to the left lower quadrant. She denied fever, chills, dysuria, hematuria, nausea, vomiting, or diarrhea. Labs again showed anemia, and urinalysis showed 2+ blood with trace bacteria. CT of the abdomen and pelvis without contrast showed continued constipation as well as free fluid around the uterus with possible hemorrhagic fluid collection. The patient was seen by gynecology and received a bowel regimen with relief of both her constipation and her pain.

The patient was discharged in stable condition and feeling well, with plans to follow up with surgery and gynecology.

## Discussion

The current report highlights a case of intraluminal appendiceal pinworms in the absence of appendiceal inflammation, characteristics that pose obstacles to the prompt diagnosis of appendicitis. This was further complicated by the incidental discovery of an ovarian cyst, which also pointed us away from an appendiceal pathology as the primary driver of the patient’s presentation. While the initially broad differential included tubo-ovarian pathology and mesenteric adenitis, histology, in this case, confirmed the diagnosis of appendiceal pinworms.

Upon resolution of the patient's symptoms following surgery, it is evident that our other differential diagnoses, such as tubo-ovarian pathology, were less likely to be driving the patient's initial presentation. Specifically, while the patient did indeed have a right-sided ovarian cyst discovered upon laparoscopy, this cyst was not large enough to be detected on CT imaging. Furthermore, upon removing the appendix, the patient’s symptoms did resolve despite surgery not having intervened on the ovarian cysts. Taken together, this suggests that the pinworm-infested appendix, rather than the cysts, drove the patient’s initial presentation and clinical deterioration.

The diagnostic workup for this case was not typical for *Enterobius vermicularis*. In a patient in endemic regions or in a child with the common presentation of pruritus ani or genitourinary infections, clinical suspicion for pinworms must be high, and the diagnosis should be confirmed with perianal swabbing or cellophane tape test. However, in this patient with few risk factors and few presenting symptoms indicative of *Enterobius vermicularis*, the workup was done for nonspecific abdominal pain, for which diagnostic laparoscopy is indicated [13]. In light of inconclusive ultrasound findings, a CT abdomen pelvis was performed, and in light of inconclusive CT findings, an exploratory laparoscopy was performed.

*Enterobius vermicularis* is the most common helminthic infection worldwide [1]. Despite the prevalence of *Enterobius vermicularis* infections in the gastrointestinal tract, infections manifesting as appendicitis are much less common, estimated to be between 1% and 4.2% [14,15]. *Enterobius vermicularis* is most common in the six- to 15-year-old age group, and its predilection for female hosts is nearly twice that of male hosts [14].

Some commonalities among cases of appendiceal *Enterobius vermicularis* include anorexia and tenderness to palpation in the right inferior fossa [16]. An absence of fever, leukocytosis, and a left shift of the white cell count may also be seen [16,17]. This suggests that appendiceal pinworms may be best delineated from acute appendicitis based on the characteristics the former lacks rather than the presence of unique clinical manifestations. It is worth noting, however, that a review of the Surgical Pathology Database at Children’s Hospital in Columbus, Ohio, found fever and leukocytosis in the majority of the symptomatic children with appendiceal pinworms [18]. Furthermore, abdominal imaging is often either negative for appendiceal inflammation or simply inconclusive [19,20]. Taken together, the available research and the current case report highlight the diagnostic challenge of elucidating *Enterobius vermicularis* as the likely etiology of a patient presenting with appendicitis.

Histological evaluation of the appendix post-appendectomy seems the most reliable way to determine the presence of pinworms. Appendiceal parasitic infections generally tend to include features of visualized helminths, lymphoid hyperplasia, and eosinophilia [15]. Surgical management of pinworms is typically not necessary, and medical management is relatively simple, typically consisting of mebendazole and albendazole [12].

## Conclusions

In sum, *Enterobius vermicularis* in the appendix can mimic the symptoms of acute appendicitis. This represents a unique challenge for the diagnosis and management of patients with appendiceal pinworms. While medical management is simple, these infections can be easily missed without post-surgical histological examination. Case reports such as these highlight the importance of recognizing pinworms as a possible etiology for presentations concerning acute appendicitis, as recognizing these cases can prevent unnecessary appendectomies.
